# Biological embedding of childhood adversity: from physiological mechanisms to clinical implications

**DOI:** 10.1186/s12916-017-0895-4

**Published:** 2017-07-20

**Authors:** Anne E. Berens, Sarah K. G. Jensen, Charles A. Nelson

**Affiliations:** 1 Boston Children’s Hospital, Boston, Massachusetts USA; 2 Harvard Medical School, Boston, Massachusetts USA; 3 Laboratories of Cognitive Neuroscience, Boston Children’s Hospital/Harvard Medical School, 1 Autumn Street, Boston, 02215 Massachusetts USA; 4 Graduate School of Education, Harvard University, Cambridge, Massachusetts USA

**Keywords:** Adverse childhood experiences, Brain development, Stress, Health promotion, Social disparities, Primary care

## Abstract

**Background:**

Adverse psychosocial exposures in early life, namely experiences such as child maltreatment, caregiver stress or depression, and domestic or community violence, have been associated in epidemiological studies with increased lifetime risk of adverse outcomes, including diabetes, heart disease, cancers, and psychiatric illnesses. Additional work has shed light on the potential molecular mechanisms by which early adversity becomes “biologically embedded” in altered physiology across body systems. This review surveys evidence on such mechanisms and calls on researchers, clinicians, policymakers, and other practitioners to act upon evidence.

**Observations:**

Childhood psychosocial adversity has wide-ranging effects on neural, endocrine, immune, and metabolic physiology. Molecular mechanisms broadly implicate disruption of central neural networks, neuroendocrine stress dysregulation, and chronic inflammation, among other changes. Physiological disruption predisposes individuals to common diseases across the life course.

**Conclusions:**

Reviewed evidence has important implications for clinical practice, biomedical research, and work across other sectors relevant to public health and child wellbeing. Warranted changes include increased clinical screening for exposures among children and adults, scale-up of effective interventions, policy advocacy, and ongoing research to develop new evidence-based response strategies.

## Background

Epidemiological studies have demonstrated that adverse childhood experiences, namely exposures such as neglect, abuse, caregiver mental illness, and family or community violence, predict poorer long-term outcomes across health and social domains. Outcomes associated with early adversity include higher risk of type 2 diabetes, obesity, ischemic heart disease, cancers, depression, addictions, and premature mortality, as well as social outcomes including unemployment and lower educational attainment [1–8]. Particularly convincing evidence comes from large birth cohorts and prospective, longitudinal life-course studies exploring predictive relationships [3, 5–9]. Meanwhile, human and animal research has provided insights into candidate molecular mechanisms by which early adversity may become “biologically embedded” in disrupted physiology [10]. Such findings support life-course models of human health describing how early physiological development interacts over time with behavior and ongoing risk environments to shape outcomes holistically [7].

Nevertheless, evidence about the pathogenic effects of childhood psychosocial adversity has not been widely applied in clinical practice or public health initiatives. Such knowledge has the potential to improve screening and intervention strategies aiming to decrease exposure to early adversity (primary prevention), limit resulting pathology (secondary prevention), and help those already suffering effects (tertiary prevention and treatment). Efforts must span the life course, involving pediatric and adult clinicians, researchers, educators, public health practitioners, families, and communities. Awareness of the effects of adversity can furthermore enhance investigations into the roots of human disease.

This review surveys the evidence on biological mechanisms thought to link early childhood adversity to later disease. While prior literature has predominantly described changes in one or a few physiological axes, this review summarizes changes comprehensively across body systems, offering a unified orientation for clinicians and researchers. The specific questions addressed include (1) How can often time-limited early exposures produce durable physiological changes? (2) How do such physiological changes converge to generate disease? (3) What factors underlie “differential susceptibility” to developmental adversity, and how can interventions promote resilience? Finally, we consider how answers to these questions should shape action across social sectors to promote child wellbeing and lifelong health.

### Defining early life adversity

In this review, we conceptualize childhood adversity as a negative childhood experience associated with increased lifetime risk of poorer health and social outcomes. The review is limited to postnatal exposures, while separate literature covers important effects of prenatal adversity [11]. We specifically consider psychosocial adversity, namely that involving relationships (to caregivers, family, community, peers) and other social experiences interacting with psychological processes [12]. Examples of psychosocial adversities include childhood maltreatment, violence exposure, caregiver psychopathology, unstable or depriving care environments (e.g., low-quality foster or institutional care), adverse societal exposures such as crime and discrimination, and other causes of psychological stress or trauma. Various childhood adversities are prevalent globally. A recent review found that at least 44% of children in developed countries and 59% in developing countries had been victims of physical, emotional, or sexual violence or had witnessed domestic or community violence in the preceding year [13]. Caregiver poor mental health is also common, with depression currently representing the leading cause of disease-related disability globally [14].

For brevity, we refer to childhood psychosocial adversity as “early life adversity” (ELA), employing an aggregative approach to conceptualize exposures. Such an approach facilitates the synthesis of complex evidence for application, and is supported by observed dose–response effects linking cumulative early adversity to later outcomes [1, 3, 5], and by the “allostatic load” paradigm exploring pathogenic effects of cumulative all-cause stress [15]. Such aggregative approaches require complementary efforts to differentiate effects of exposures varying in nature, timing, and intensity [16]. Here, we do not specifically examine low childhood socioeconomic status (SES) as a psychosocial adversity, as poverty influences health in part via non-psychosocial pathways (e.g., increasing exposure to physical environmental hazards). Meanwhile, some families living in poverty provide safe psychosocial environments despite the challenges posed by socioeconomic disadvantage. Nevertheless, childhood adversities are strongly partitioned by SES, and shaped by inequities intertwined with poverty such as those defined by race, gender, immigration status, class, and other axes of social inequality.

### Biological embedding

Biological embedding describes processes by which initially transient, homeostatic responses durably alter physiology [10]. Events early in life may be embedded preferentially due to a preponderance of sensitive periods, or windows of rapid development and heightened plasticity (responsiveness to experience). While traditionally described in neurodevelopment [16], sensitive period effects have been suggested elsewhere, including in the immune [17] and metabolic [18] systems. Epigenetic processes represent a key family of mechanisms driving embedding. Epigenetic change involves stable alteration of gene expression via mechanisms including, among others, attachment of chemical residues (e.g., methyl groups) to DNA or to molecules involved in packaging and transcriptional control (e.g., histones) [19].

### Methodological challenges

A key methodological challenge is the difficulty of causal demonstration amidst social complexity. While epidemiological studies statistically explore confounding and mediational pathways, randomized controlled trials – the “gold standard” in causal inference – are often impossible or unethical. This challenge necessitates substantial use of animal models, enabling controlled experimentation and use of targeted molecular manipulations clarifying causal pathways. These models are considered in this review when potentially useful to understand human processes. An additional challenge has been the reliance on retrospective self-reporting of ELA in many studies. Such reports may agree only moderately with prospective measures, and could be more prone to bias, though both types of measures tend to predict similar disease and social outcomes [20]. We therefore focus on the direction (versus size) of effects and on physiological mechanisms, and prioritize studies using prospective, longitudinal designs.

### Search strategy

We identified peer-reviewed, academic literature from multiple databases, including PubMed, Medline, and PsycINFO, using search terms specifying timing in early life (e.g., early, child*, infan*) and adverse exposures (e.g., advers*, psychological stress, maltreat*), as well as terms for specific physiological axes as appropriate. Priority was given to more recent studies, major reviews, and prospective human studies. Cross-sectional and animal studies were included where prospective human evidence was unavailable.

## Biological embedding by physiological axis

**Fig. 1:**
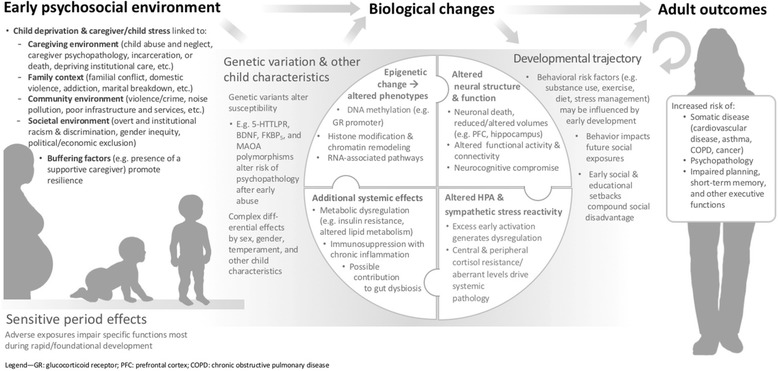
Conceptual model of the biological embedding of early psychosocial adversity. Adapted from [113]

ELA has diverse effects across neural, endocrine, immune, metabolic, and gut microbial axes, as reviewed below. Table 1 summarizes key findings, while Fig. 1 provides a working conceptual model of ELA’s biological embedding.

**Table 1:** Selected effects of early life adversity (ELA) on physiological functioning

Examples of physiological changes observed after ELA	Overall clinical and functional effects	Key reviews
**Brain structure and activity**		
Structural variation in gray and white matter	Increased risk of: - Impairments in executive functioning (e.g., working memory, cognitive control) - Impaired emotion regulation and social functioning - Adverse effects on reward processing and stress regulation (e.g., hippocampus, amygdala, PFC) may increase risk of mood and substance use disorders	Bick & Nelson, 2016 [21] Hart & Rubia, 2012 [24] McEwen, 2013 [50] Nemeroff et al., 2016 [25]
1) Changes in local/global gray matter volumes a) Some evidence for widespread, global gray matter change b) Decreased gray matter volume of PFC and hippocampus c) Complex volumetric changes in amygdala		
2) Changes in local/global white matter volume and microstructure		
a) Complex white matter volumetric changes in frontal lobes b) Microstructural variation in various white matter tracts that may impair communication between brain regions		
Functional variation in brain activity and functional connectivity		
3) Aberrant amygdala reactivity to emotional stimuli		
4) Alterations in amygdala-PFC connectivity		
Altered neurotransmitter metabolism or production		
5) Potential altered neurotransmitter levels/signaling involving key molecules, e.g., serotonin, dopamine, GABA, glutamate		
**Neuroendocrine (HPA) stress response axes**		
Hyper-responsiveness	- Both HPA hyper- or hypo- reactivity are characteristic patterns generating excess “allostatic load,” linked to cardiovascular disease, metabolic syndrome, accelerated cellular aging, and various psychopathologies - Downstream effects of aberrant cortisol levels (e.g., neurotoxicity, heightened inflammation, metabolic dysregulation) may drive pathology across other axes	Doom & Gunnar, 2015 [36] Heim & Binder, 2012 [87]
1) Enhanced ACTH and cortisol response to stress/stimulation		
2) Evidence of impaired GR-mediated feedback inhibition		
Hypo-responsiveness		
4) Blunted HPA response (ACTH and cortisol) to stress/stimulation		
5) Heightened ACTH response with inappropriately blunted cortisol (normal or low)		
Altered basal diurnal rhythms		
3) Elevated, or suppressed, average cortisol/CRF		
6) Complex changes to diurnal cortisol rhythms (e.g., lower morning and flatter decline, or higher morning and steeper decline)		
**Autonomic functioning**		
1) Complex patterns of sympathetic- or parasympathetic-predominant imbalance of reactivity to acute stress, with alterations in responsiveness and counter-regulatory control	- Both parasympathetic- or sympathetic-predominant autonomic imbalances are linked to diseases of elevated “allostatic load” (discussed above)	Alkon et al., 2012 [55] El-Sheikh et al., 2009 [56]
2) Elevated or decreased sympathetic or parasympathetic basal tone		
**Immunity and inflammation**		
1) Systemic immune suppression (e.g., impaired cellular immunity)	- Chronic inflammation linked to increased cardiometabolic and other disease risk - Immunosuppression linked to impaired control of infectious/neoplastic threats	Slopen et al., 2012 [66] Baumeister et al., 2016 [67]
2) Chronic basal inflammation (e.g., elevated CRP, TNF- α, IL-6) 3) Heightened inflammatory reactivity		
**Metabolism**		
1) Impaired peripheral glucose handling with insulin resistance	- Heightened risk of type 2 diabetes, obesity, hyperlipidemia, or other metabolic disease	Maniam et al., 2014 [70]
2) Altered fat metabolism with dyslipidemia		
**Microbiome functioning (emergent evidence, animal models only to date)**		
1) Transient microbiome perturbations after stress in infancy linked to aberrant immune development	- May contribute to inflammation, immune-suppression, and/or neurodevelopmental risk	O’Mahony et al., 2015 [74]
2) Possible durable microbiome changes in adults after early stress		

*PFC* prefrontal cortex, *ACTH* adrenocorticotropic hormone, *GR* glucocorticoid receptor, *CRF* corticotropin releasing factor, *CRP* C-reactive protein, *TNF* tumor necrosis factor, *IL-6* interleukin-6, *HPA* hypothalamic-pituitary-adrenal

### Axis 1: The brain

Human brain maturation is a protracted process beginning in fetal life and continuing into early adulthood [21]. Dramatic growth in gray and white matter occurs in the first 2 years of life, when the brain attains 80–90% of its adult volume before continuing to grow at an attenuated rate [22, 23]. Alongside growth, experience-dependent neural pruning eliminates inactive synapses. Anatomically, the brain matures “from the bottom up,” beginning with primitive brainstem structures and progressing anatomically in anterior-posterior and inferior-superior directions, culminating with the prefrontal cortex (PFC). Functional development similarly progresses from basic sensory and motor capacities to subsequent language and executive functioning (e.g., cognitive control, working memory), and ultimately higher cognition [16]. Normative neurodevelopment thus enables environmental adaptation and progressively complex cognition, but leaves the brain susceptible to negative exposures for an extended period of time.

Extensive literature links ELA to pervasive, quantifiable variation in brain structure and function [15, 21, 24, 25]. Investigation has preferentially examined “stress sensitive” areas dense with glucocorticoid receptors, including limbic structures (e.g., hippocampus and amygdala) key to memory, learning, and emotion regulation, as well as the PFC, critical for higher cognition, executive functioning, and “top-down” control of lower regions [26]. Studies of adolescents and adults provide consistent evidence of smaller PFC gray matter volumes after ELA, paralleling findings from experimental animal models designed to demonstrate causality [21, 24, 25]. Smaller hippocampal volumes have been consistently observed in ELA-exposed adults, though not children, reflecting potential latent effects on a slow-developing structure. Amygdala volumetric effects are complex, including both increases and decreases, likely moderated by exposure timing and type [21, 27].

Considering potential embedding mechanisms, the “neurotoxicity hypothesis” posits that early elevation of stress mediators, particularly glucocorticoids, kills or impedes growth of neurons in stress-sensitive regions via mechanisms including oxidative damage [28]. Stress mediators potentially linked to neurotoxicity in humans include cortisol as well as inflammatory cytokines, excitatory amino acids (e.g., glutamate), and various other molecules (e.g., brain-derived neurotrophic factor (BDNF) and endogenous opioids) [29]. Oxidative stress during early neurodevelopment may also disrupt (delay or extend) neural sensitive periods [30]. Considering epigenetics, experimental animal models show altered expression of genes implicated in basic neurodevelopmental processes (e.g., cell adhesion, sensitive period closure) [31]. Human studies of ELA show genome-wide methylation changes as well as gene-specific effects on neural signaling molecules important to psychological health and neural function, for instance serotonin, glutamate, dopamine, catechol-O-methyl transferase (COMT), and BDNF [19].

Beyond excess stress, environmental deprivation may also play a role in neurodevelopmental compromise, for instance, among children in low-quality institutional care [32]. Broadly, absence of normative psychosocial stimuli (e.g., language exposure or caregiver interaction) during experience-dependent development is proposed to promote excessive synaptic pruning [33]. Indeed, children raised in depriving institutions in infancy show globally decreased cortical thickness [34], a finding possibly paralleled by reduced brain-wide dendritic arborization, spine density, and brain volume in rodent models of early deprivation (e.g., rearing in single-occupancy cages) [35]. Nevertheless, “depriving” exposures (such as caregiver absence) generally evoke potent stress responses [36] while stress mediators regulate synaptic plasticity [37], complicating efforts to discern whether the observed structural changes reflect excess pruning (versus, for instance, glucocorticoid neurotoxicity) and if these mechanisms are, in fact, independent of stress-mediated pathways.

The neurodevelopmental changes described may have far-reaching functional and health implications. Studies suggest that neural-structural changes mediate ELA effects on depression [38], while sensitive period disruption may contribute to schizophrenia and autism pathogenesis [30, 39]. Studies of ELA-associated brain functional changes show deficits in processes including emotion regulation, fear learning, and executive functioning [21]. Functional MRI studies show differences in centrally-driven reward processing that could mediate ELA-related risk of psychopathologies and substance use-related illnesses [40, 41]. Finally, disruption of central stress-regulatory structures may promote neuroendocrine disruption linked to diseases of excess allostatic load [42], as discussed below.

### Axis 2: Neuroendocrine stress regulation

ELA broadly impacts stress reactivity as controlled by the hypothalamic-pituitary-adrenal (HPA) and autonomic (sympathetic/parasympathetic) axes. Both axes are under central control by corticolimbic structures, including the PFC, hippocampus, and amygdala [29], and involve common molecular mediators (e.g., corticotropin-releasing factor (CRF), an HPA hormone and autonomic neurotransmitter) [43], suggesting potentially overlapping embedding pathways.

#### HPA axis

In response to stress, hypothalamic CRF stimulates pituitary adrenocorticotropic hormone (ACTH) release and, in turn, adrenal cortical secretion of glucocorticoids—principally cortisol in humans and corticosterone in many animal species. Glucocorticoids trigger diverse systemic homeostatic responses while exerting negative feedback on the axis. In human studies and animal experimentation, ELA consistently predicts HPA dysregulation generally persisting into adulthood, including patterns of hyper-reactivity, suggesting potential acquired resistance to glucocorticoid negative feedback [29], or hypo-reactivity, suggesting possible attenuated stress sensitivity or exaggerated axis suppression [44]. Differential patterns of dysregulation may reflect variation in factors including timing and type of ELA [45], genotype [46], current age [29], and concurrent psychopathology [47]. Importantly, HPA hyper- and hypo-reactivity both represent prototypical patterns associated with excess allostatic load, and both predict human stress-related chronic illnesses, including cardiovascular, metabolic, and psychiatric diseases linked epidemiologically to ELA [15, 29, 48]. Glucocorticoid dysregulation may also promote oncogenic tumor cell microenvironments (in part via pro-inflammatory effects, as discussed below), fostering growth, migration, invasiveness, and angiogenesis [49], thus potentially contributing to observed links between ELA and cancers [7].

Considering potential mechanisms of HPA changes, animal models of early stress have demonstrated altered expression of the glucocorticoid receptor (GR) (involved preferentially in axis downregulation) and receptors for CRF, ACTH, and other key molecules [50]. In particular, altered serotonin signaling in rats receiving unfavorable maternal care has been shown to induce hypermethylation (silencing) of the GR promoter and related genes [51]. Similar GR hypermethylation was subsequently demonstrated in hippocampal tissue [52] and peripheral lymphocytes [53] of humans maltreated in childhood. Other epigenetic changes shown in animals include genes controlling other key stress-related receptors (e.g., for CRF) and hormones (e.g., CRF, AVP, ACTH, and cortisol), as well as in neurotransmitters/neuropeptides in stress-regulatory brain regions [54].

#### Autonomic axis

In response to stress, amygdala signaling initiates sympathetic activation via the brainstem, terminating in adrenergic signals to end organs (e.g., liver, heart, digestive tract, and pancreas) and induction of adrenal medullary epinephrine/norepinephrine release producing the prototypical “fight or flight” response. The parasympathetic branch exerts countervailing control, and dynamic sympathetic-parasympathetic balance shapes overall stress physiology [55]. Experimental animal models and observational human studies have consistently linked ELA to autonomic dysregulation, including both hyper- and hypo-responsiveness of sympathetic or parasympathetic pathways. Imbalance in either sympathetic- or parasympathetic-dominant directions again represent manifestations of excess allostatic load and predict stress-related diseases, including heart disease, obesity, type 2 diabetes, cancers, and psychopathologies [55]. Pathology associations may differ by pattern of autonomic imbalance. Several studies, for instance, found that attenuated sympathetic reactivity correlated with antisocial behavior with callous-unemotional traits in ELA-exposed boys, while heightened reactivity correlated with antisocial behavior without callous-unemotional traits [56]. Such findings remain exploratory, and the direction of causal links, if present, is unclear. Among few studies specifically examining mechanisms of autonomic changes, one found that volumetric changes in the amygdala, hippocampus, and PFC statistically mediated autonomic changes as well as risk of psychopathology [57]. Overlapping regulation by corticolimbic structures and core molecular mediators (e.g., CRF) suggests that some HPA-related alterations may also impact autonomic functioning.

### Axis 3: Immune functioning

Innate and adaptive immune responses work jointly to control exogenous (e.g., microbial) and endogenous (e.g., necrotic/neoplastic) threats in processes dependent upon inflammatory mediators. When chronically elevated, however, inflammatory mediators contribute to immunosuppression as well as oxidative stress and cytotoxicity [58]. ELA has been linked in human studies and animal experimentation to chronic inflammation [59] and low-level immunosuppression, including impairment of mucosal immunity in children [60] and cellular immunity (e.g., poorer control of latent viral infection) in adolescents [61] and adults [62]. Important work has characterized a "pro-inflammatory phenotype", involving exaggerated cytokine response to bacterial challenge and progressive glucocorticoid receptor desensitization, among ELA-exposed individuals [63]. Considering potential mechanisms, acquired peripheral glucocorticoid resistance may attenuate cortisol’s anti-inflammatory effects [18]. Meanwhile, genome-wide analysis in ELA-exposed individuals has shown increased expression of genes controlling not only cortisol output, but also the activity of key inflammatory mediators like NF-κβ and interleukin-6 (IL-6) [64], with potential antecedents including developmental programming of monocytes for excessive inflammatory responses [18, 65]. Finally, emerging research posits that ELA-related gut dysbiosis may contribute to chronic inflammation, as discussed below.

Health implications of immunosuppression include compromised control of infection and other threats. Meanwhile, inflammatory mediators linked to ELA (e.g., IL-1, IL-6, TNF-alpha, CRP, and fibrinogen) are implicated in risk of cardiovascular and metabolic disease [17, 66, 67]. Inflammation is also a proposed mechanism mediating ELA effects on later depression, age-related diseases [3], neurodevelopmental changes [40], cancers [49], and other systemic effects discussed. Considering cancer risk in particular, immunosuppression impairs control of latent oncogenic viruses [68], while inflammation further promotes oncogenic tumor microenvironments in conjunction with stress mediators, as discussed above [49].

### Axis 4: Metabolic health

Interest in metabolic embedding of ELA stems from epidemiological [1, 69] and clinical [70] studies linking ELA to obesity, dyslipidemia, and type 2 diabetes, raising questions about possible causal pathways. While research directly linking ELA to altered development of metabolic physiology remains emergent (versus clear indirect impacts via, e.g., chronic inflammation [3]), potential loci of embedding are multiple. Feeding-related regulation involves, among other networks, dopaminergic reward pathways under top-down control by the PFC, and hypothalamic nuclei integrating nutrient signals to induce hunger or satiety, and systemic shifts between catabolism and anabolism [71]. Peripheral energy homeostasis involves an interplay of anabolic (e.g., insulin) and catabolic (e.g., cortisol, glucagon, epinephrine/norepinephrine) signals promoting increased glycemia and tissue insulin resistance.

Considering mechanisms of potential ELA effects, chronic inflammation, as well as excess catabolic signaling in those with hypercortisolemia, are proposed to drive metabolic dysfunction. Preliminary models also posit that ELA may durably alter hepatic expression of cortisol-activating and -metabolizing enzymes, enhancing tissue-level insulin resistance even in those who later suppress hypercortisolemia [70]. Furthermore, a previous study linked ELA to altered central reward processing promoting excess food intake in some individuals [72]. Additional work is needed to explore the hypothesized pathways.

### Axis 5: The microbiome

The gut microbiome represents the collective genome of nearly 100 trillion commensal microorganisms, including over 1000 bacterial species. Dysbiosis, a pathogenic disruption of gut microbial composition or host-microbe interactions, is implicated in diseases including obesity, type 2 diabetes, and depression [73]. While genetically influenced, gut microbial composition responds to factors including stress, diet, infection, drugs, and toxins, making the gut a potential mediator between environment and disease. Various previous studies have suggested profound microbiome effects on neuroendocrine and immune function, such that dysbiosis could compound ELA-related changes including cortisol dysregulation and chronic inflammation [73–77]. Furthermore, growing literature on the “gut-brain axis” describes microbial influence on neural development and functioning [78]. Pathways of influence may include microbial vagus nerve activation, neural signaling by microbial metabolites or molecular patterns, heightened inflammation with downstream neural effects, and induction of epigenetic changes [77, 79, 80]. In animal experimentation and some small human studies, dysbiosis has also been shown to impact relevant brain and behavioral parameters, including cortisol regulation, depressive and anxious symptomatology, and social functioning [77, 79].

Whether ELA itself produces dysbiosis is a question of ongoing interest [74]. A study in rodents found that infant maternal separation durably altered fecal microbiota and increased later inflammatory reactivity [81]. Work in monkeys, meanwhile, found that transient dysbiosis triggered by infant maternal separation predicted durable immune dysfunction, supporting the possibility of early microbiome effects on development in other axes [82]. If human research replicates such findings, the health implications may be considerable.

## Interactive effects across axes

The above evidence illustrates how ELA-related physiological changes generate feed-forward synergies; for instance, if glucocorticoid toxicity compromises brain regions tasked with stress regulation [29], or stress-related inflammation further disrupts neural, gut microbial, and metabolic axes to compound HPA dysregulation and further inflammation [83]. Meanwhile, brain functional changes (e.g., altered executive functions and reward processing) may shape health-related behaviors and ongoing social risk exposures [84]. Synergistic effects of ELA thus produce wide-ranging physiological changes marked by aberrant neural function, endocrine activity, chronic inflammation, immunosuppression, insulin resistance and, potentially, dysbiosis. These changes are substantially mediated by altered development of stress-response systems; when acute, activation of these systems generates adaptive changes across body systems (e.g., immune, metabolic, cardiovascular) to address threats. However, chronic or excessive activation contributes to the pathogenic physiological “wear and tear” described within the allostatic load paradigm [15, 29]. In full, ELA-induced changes may mediate epidemiological links to key diseases, including, among others, obesity, dyslipidemia, type 2 diabetes, atherosclerosis, asthma, thromboembolic events (myocardial infarction, stroke), cancer onset and progression, as well as addictions, psychopathology, and adverse social outcomes [1–6, 18].

## Differential susceptibility to adversity

Despite described trends, outcomes among ELA-exposed individuals are markedly diverse. A rich literature describes this apparent differential susceptibility to adversity, as selectively reviewed in Table 2 and recommended as further reading [85, 86]. Some observed modifiers of ELA effects include genetics [25, 87–89], child sex and/or gender [19, 90, 91], exposure features (e.g., timing, nature, and intensity) [21, 25], and the presence of other risks or protective factors [36]. Of note, substantial literature suggests that nurturing caregiving is a particularly powerful protective factor mitigating ELA associations with physiological parameters, including elevated allostatic load [92, 93], inflammation [94], cortisol reactivity [95], and cellular aging [96]. Considering neurodevelopment, a prospective study found that caregiving behaviors mediated the association of early childhood socioeconomic stress with hippocampal volumetric change [97]. Such studies suggest that caregiving quality critically shapes psychosocial risk trajectories and developmental effects.

**Table 2:** Selected effect modifiers

Modifier	Examples of findings	Further reading
Genetic variability	• Genetic polymorphisms found to moderate associations between ELA and various outcomes; Specific examples of outcomes impacted with implicated genes include:	
	o Emotional and neuroendocrine stress reactivity: 5-HTTLPR	Lester et al., 2006 [86]
	o Inflammatory response to stress: 5-HTTLPR	Fredericks et al., 2010 [88]
	o Common forms of psychopathology, including depression, ADHD, and substance addiction: NR3C1, CRHR1, OXTR, 5-HTTLPR, HTR3A, DRD2, MAOA, BDNF, COMT o Atherosclerosis risk: MAOA	Nemeroff et al., 2016 [25] Heim & Binder, 2012 [87] Zhao et al., 2013 [89]
Child sex and gender	• Complex sex differences in HPA and autonomic dysregulation after early stress observed in animals and humans	Essex et al., 2013 [19]
	• Differential effects of maternal vs. paternal stress on boys vs. girls leads some to posit ELA effect moderation by socially embedded gender roles	
	• Genetic moderators of the effects of ELA may be sex and/or gender specific o Meta-analysis found stronger effect of MAOA genotype on psychopathology in boys o Different polymorphism on the 5-HTTLPR gene have been linked with increased risk of depression following ELA in males vs. females	Kim-Cohen et al., 2006 [90] Brummet et al., 2008 [91]
Other child characteristics	• Pre-existing health conditions, e.g., prematurity, poor physical health status, etc. alter social and physiological consequences of ELA	Doom & Gunnar, 2015 [36]
	• Child temperament, sensitivity to the environment, and emotion processing are associated with risk for psychopathology and may affect the ways in which children respond to adversity	Lester et al., 2006 [86]
Exposure characteristics	• Characteristics of the exposure, including type (e.g., sexual, physical, emotional abuse, or neglect), chronicity, and intensity, modify associations with physical and mental health outcomes	Nemeroff et al., 2016 [25]
	• Exposures occurring during early sensitive periods can have heightened impacts on specific developmental domains leading to “timing effects”	Bick & Nelson, 2016 [21]
Social context and caregiving	• Family structure and stability, birth order, caregiver stress and social support, community and societal context may modify effects of specific adversities	Doom & Gunnar, 2015 [36]
	• Presence of a dependable, supportive caregiver may “buffer” children from effects of otherwise adverse environment	
Cumulative occurrence	• Dose-response relationship between number of adversities and health and social effects are observed in large epidemiological studies	Felitti et al., 1998 [1] Danese et al., 2009 [3]

*ADHD* attention deficit hyperactivity disorder, *HLA* hypothalamic-pituitary-adrenal, *ELA* early life adversity

## Clinical, research, and public health applications

The evidence linking ELA to lifelong health is substantial, with important implications for clinical practice and public health summarized in Table 3. We highlight four recommendations in particular. First, we suggest that screening for ELA should become a routine part of clinical care for children and adults. This aspect of the “developmental history” can provide information about a patient’s risk of major pediatric and adult diseases, facilitating social support, protective intervention, and/or decisions about disease screening and prevention.

**Table 3:** Proposed clinical implications of reviewed findings

Practitioner activity	Recommendations	Recommended resources
Understanding disease etiology and risk	Consider how ELA contributes to a patient’s risk of common health problems, e.g.: • Mental health disorders: Depression, anxiety, substance use disorders, post-traumatic stress disorder, psychosis • Cardiovascular disease: Ischemic heart disease, hypertension, atherosclerosis • Metabolic pathology: Obesity, type 2 diabetes, dyslipidemia, metabolic syndrome • Neoplasm: Breast, liver, lung cancers	Results of major epidemiological studies assessing health effects of ELA [1–6] Further reading suggested throughout
Screening	• Screen for ELA history • Assess social service and protection needs • Consider ELA history when assessing risk and screening for ELA-related diseases or developmental needs	Adverse Childhood Experiences Questionnaire [1] WHO Adverse Childhood Experiences International Questionnaire [104] American Academy of Pediatrics Resilience Project Clinical Screening Tools [105]
Intervention	*General practice* Provide access to: • Mental healthcare • Early prevention and treatment for other ELA-related diseases • Social services and poverty alleviation • Violence response and prevention interventions *Pediatric practice* • Family and caregiver support programs • Early development interventions • Services to prevent or respond to ELA exposures, including child protection services	WHO Preventing Child Maltreatment guide [106] WHO mhGAP Intervention Guide [107] Interventions resources to support healthy child development from Frontiers of Innovation – Center on the Developing Child at Harvard University [108]
Transforming care models	Adopt best-practices from “medical home models” to support ELA-exposed patients, including strategies promoting: • Patient- and family-centered wraparound care • Cultural competency • Enhanced access and follow-up	National Center for Medical Home Implementation Tools & Resources [109]
Advocacy	Incorporate evidence on ELA into advocacy relating to: • Access to mental health services • Poverty alleviation, criminal justice reform, and violence prevention • Fair parental leave and high-quality child care • Immigration and refugee policies protecting children and families	WHO guidance package on Advocacy for Mental Health [110] United Nations Children’s Fund policy advocacy and children's rights tools [111] Children’s Defense Fund policy campaign resources [112]

*ELA* early life adversity, *WHO* World Health Organization

Second, screening for ELA must be matched by investment in scale-up of known effective interventions promoting health by addressing ELA. Considerable evidence suggests that caregiving-focused interventions, for instance, may mitigate the physiological effects of ELA. Some parameters improved by caregiving-focused interventions in longitudinal research include ELA-associated chronic inflammation [98], telomere shortening (accelerated genetic aging) [99], and gray matter volumetric changes [100]. Similarly, cortisol reactivity appears to be sensitive to caregiver-targeted interventions and to psychological support interventions with ELA-exposed individuals [101]. Scale-up investments must include quality monitoring and ongoing assessment of impact at scale. Assessments must disaggregate effects by population subgroups, for instance, as defined by culture, SES, religion, race, or ethnicity, to identify diverse needs [102].

Third, investigators must continue to test new intervention strategies to prevent or reduce the physiological effects of ELA. New approaches should be ever more accurately targeted (e.g., based on genotype-dependent response variation), scalable, effective, and evidence based, making use of the rich literature on biological embedding. In particular, novel approaches are needed to reach the most vulnerable families often least impacted by existing strategies [102]. Efforts should be aided by ongoing development of biomarkers of ELA [103], which can be used to track intervention effects and optimize timing and targeting. Additional research priorities include better characterization of ELA-microbiome links, and consistent use of prospective ELA measures.

Finally, we recommend that practitioners across multiple social sectors recognize ELA as a common soil giving root to various manifestations of poor health over the life course, and better align strategies to advance child welfare and public health. Disease prevention paradigms must move beyond proximal focus on risk behaviors (e.g., diet, substance use) for specific diseases towards life-course models accounting for early influences on lifelong health. Efforts require coordination across health, social services, education, justice, child protection, and other sectors to improve alignment around children’s needs. Among others, relevant priorities might include improving access to mental health services, childcare, and parental leave, expanding family poverty programs, seeking immigration and criminal justice practices that avoid separating children from nurturing caregivers, and addressing racial inequities impacting children.

## Conclusions

The findings reviewed here explore various biological mechanisms that may explain links between adverse childhood experiences and disease. These insights can inform efforts to improve health across the life course. As the emergence of novel tools, such as biomarkers of early adversity, drives a new wave of intervention research, strong collaboration is needed between medical and public health practitioners, families, and communities based on a deep appreciation for the effects of early adversity. The understanding of the physiology of biological embedding, as explored here, supports those leading practice-transforming efforts.
